# Association of Depression and Anxiety With Hypertensive Crisis: A Cross-Sectional Study From a Hospital Setting in Karachi, Pakistan

**DOI:** 10.7759/cureus.29792

**Published:** 2022-09-30

**Authors:** Fatimah S Yousuf, Aiman Arif, Raheela Bibi, Aysha Almas

**Affiliations:** 1 Department of Medicine, Aga Khan University Hospital, Karachi, PAK

**Keywords:** association, depression in elderly, anxiety, depression, hypertensive crisis

## Abstract

Introduction

Hypertension, a leading risk factor for cardiovascular death, has been closely linked with depression and anxiety. The aim of this study was to examine the association of depression or anxiety with hypertensive crisis in patients and also see if the association is affected by age group or gender. This was carried out in a hospital setting.

Methods

This cross-sectional study was conducted between July 2019 and March 2022 on 290 patients admitted to the Aga Khan University Hospital (AKUH), Karachi, Pakistan. All adult patients more than 18 years of age admitted with uncontrolled hypertension with a systolic blood pressure of >140 and a diastolic blood pressure of >90 admitted through emergency were included. A hypertensive crisis was defined as a systolic blood pressure greater than 180 mm Hg or a diastolic blood pressure greater than or equal to 120 mm Hg, with or without accompanying end organ damage. Symptoms of depression and anxiety were evaluated using the Hospital Anxiety and Depression Scale (HADS), with a cut-off score ≥8.

Results

Of the patients identified with uncontrolled hypertension, a total of 140 (48.3%) of the patients presented with a hypertensive crisis, while 150 (51.3%) did not have a hypertensive crisis at presentation. In the hypertensive crisis group, 60 (49.3%) had HADS scores consistent with depression, while 83 (59.3%) had HADS scores consistent with anxiety. In patients with hypertensive crisis, HADS depression and anxiety were most prevalent in the 61-75 age group (39.7%). In the comparison of gender, it was found that males and females with hypertensive crisis had an almost equal prevalence of anxiety (49.4% in males versus 50.6% in females). A slightly higher prevalence of depression was seen in females with hypertensive crises when compared to males.

Conclusion

We found no association between depression or anxiety with hypertensive crisis, and the association is not affected by age group or gender. However, do note that half of the patients with hypertensive crises had depression or anxiety. Future large multicentered studies are required to study the link in greater detail.

## Introduction

Worldwide, hypertension is the leading risk factor for cardiovascular disease [1,2]. Estimates suggest that 31.1% of adults (1.39 billion) worldwide had a diagnosis of hypertension in 2010. The prevalence of hypertension among adults was higher in low-and middle-income countries (LMICs) (75%, 1.04 billion people) than in high-income countries (25%, 349 million people) [3]. In Pakistan, which is considered to be an LMIC, a nationwide survey conducted in 2015-2016 showed a 46% prevalence of hypertension [4]. Besides the well-known chronic effects of hypertension on the cerebral, renal, and cardiovascular systems, uncontrolled hypertension can also precipitate an acute and severe elevation in blood pressure termed a 'hypertensive crisis', which is a systolic blood pressure of greater than or equal to 180 mm Hg or a diastolic blood pressure of greater than or equal to 120 mm Hg. It is further categorized into hypertensive urgency or emergency, depending on the presence or absence of end-organ damage [5]. It is estimated that approximately 1-2% of the population with hypertension will present with this potentially life-threatening complication [5].

While hypertension is the leading risk factor for cardiovascular diseases resulting in high mortality, depression is a common psychiatric illness resulting in a high rate of disability-adjusted life-years (DALYs), which quantifies health loss due to specific diseases and injuries [6]. Between 2007 and 2017, mental disorders resulted in significant increases in DALY counts, accounting for 16,800 DALYS (in thousands) in males and 26,300 DALYS in females in 2017 [6]. According to the Global Burden of Diseases, Injuries, and Risk Factors Study (GBD) 2019, depression ranked second among the top 25 leading causes of mortality years lived with disability (YLD) and was ranked highest among psychiatric disorders. Depression was also reported to be the seventh leading cause of total disability-adjusted life years (DALY), which reflects the significant disease burden of this disorder [7]. In Pakistan, the mean overall prevalence of anxiety and depression has been found to be 34% [8], which is significantly higher than the global lifetime prevalence of 8-12% [8,9].

Literature reports on the association between hypertension and depression [8]. We have previously reported a prevalence of 56.3% of depression among patients with uncontrolled hypertension, also consistent with the findings by Wang et al. [10,11]. However, the role of depression in a hypertensive crisis is an area less studied. The underlying mechanisms of the link between depression and hypertensive crisis could be attributed to different mechanisms such as endothelial dysfunction, altered hypothalamic-pituitary-adrenal axis, or autonomic dysfunction with sympathetic activation [11]. Neurotransmitters involved in depression such as serotonin and catecholamines like norepinephrine can have a major impact on the peripheral cardiovascular system, including short-term and long-term blood pressure regulation [12].

With the rising burden of hypertension and psychiatric illnesses, including depression and anxiety, it is important to find out if there is any existing link between the two and also identify the current burden in this high-risk population for cardiovascular diseases. As the hypertensive crisis is an urgent and emergent situation, most of this patient population can only be accessed through secondary and tertiary care hospitals in a LMIC like Pakistan. The aim of this study was to delineate the association of depression or anxiety with hypertensive crisis in patients from a hospital setting and also see if the association is affected by age groups or gender.

## Materials and methods

This cross-sectional study was conducted between July 2020 and March 2022 on patients admitted to the Aga Khan University Hospital (AKUH). AKUH is one of the largest Joint Commission International Accredited (740 bedded) tertiary care university hospitals in the country and caters to a variety of patients referred from all over Pakistan. All adult patients more than 18 years of age admitted with uncontrolled hypertension with a systolic blood pressure of >140 and a diastolic blood pressure of >90 admitted through emergency were included. Those who were on anti-depressants or had low GCS (Glasgow Coma Score) were excluded from the study.

As established in the literature, the hypertensive crisis was defined as a systolic blood pressure of more than 180 mm Hg or a diastolic blood pressure of greater than or equal to 120 mm Hg, with or without accompanying end-organ damage [5]. At least two readings were taken in the emergency room to document the hypertensive crisis. Anxiety and depression were assessed using the Hospital Anxiety and Depression Scale (HADS), widely used as a tool for assessing psychological distress in patients and nonclinical groups [13]. We chose to use the HADS scale since it has the advantage of detecting both depression and anxiety in patients with comorbid physical conditions, as it excludes somatic elements [14]. It is a validated tool used in various populations and has been translated into at least 30 different languages. As validated by a systemic review and meta-analysis of 747 studies, it also has good internal reliability [14,15].

Those patients with a history of anxiety or depression for at least six weeks prior to presentation were assessed through HADS. The HADS is a 14-item scale (7 for anxiety and 7 for depression) that requires respondents to give a verbal response that is scored as an index of the severity of anxiety or depression. Each item is scored using a four-point Likert scale, with the scoring different for each item. The scores are then calculated to produce two subscales corresponding to anxiety (HADS-A) and depression (HADS-D) separately (each out of 8) [16]. The diagnosis of anxiety or depression is made when a patient scores 8 or more on the HADS. This has also been validated in Urdu in Pakistan [17] and has been used in clinical settings [18].

The covariates in the study included age, gender, education, employment status, marital status, obesity, diabetes mellitus, coronary artery disease, adherence to an antihypertensive regimen, alcohol abuse, smoking, duration of hypertension, and a history of cerebrovascular disease. According to the published guidelines, diabetes was diagnosed if the patients were previously known diabetics or had a random plasma glucose value of ≥200 mg/dl (≥11.1 mmol/l) or had a fasting plasma glucose value of ≥126 mg/dl (≥7.0 mmol/l) or had an HbA1c ≥ 6 [19], whereas coronary artery disease was considered to be present in patients who had any history of stable ischemic heart disease or acute coronary syndrome (ST-elevation myocardial infarction [STEMI], non-ST-elevation myocardial infarction [NSTEMI], or unstable angina) [20]. With regards to addiction, alcohol abuse was considered to be present if the daily alcohol consumption exceeded three drinks a day (corresponding to ~30 g/day) [21]. On the other hand, past smokers were defined as subjects who had stopped smoking at least one year ago. Current smokers were further categorized according to the number of cigarettes smoked per day (light = 1 to 9, moderate = 10 to 19, heavy = more than 20) [22]. Cerebrovascular disease was considered to be present in those with a history of neurological symptoms or symptom complexes caused by cerebral ischemia or hemorrhage [23]. According to body mass index (BMI) values for the Indo-Asian population, a BMI of more than 23 was used to define obesity, and subjects with a BMI of less than 23 were regarded as non-obese [24].

A sample size of 290 participants was targeted to estimate the prevalence of depression or anxiety at 48.6% and a significance level of 0.05. The sample size estimate was based on using a power of at least 80%.

Statistical Package for Social Sciences (SPSS version 19.1®, International Business Machines Corporation (IBM), Armonk, NY) was used for analysis. The mean and standard deviation were calculated for quantitative variables and frequency and percentage for qualitative variables. For each individual item question on the HADS-A and HADS-D, a numerical code was assigned according to scale. All values for the 14-item questions for HADS-A and HADS-D were calculated. The sum of HADS-A and HADS-D was then categorized as >8 and <8. A score of >8 on either scale was suggestive of having anxiety or depression or both. Comparison between qualitative variables was made using chi-square tests and multivariate logistic regression was used to calculate the adjusted odds ratio and 95% confidence intervals. A p-value <0.05 was considered to be significant.

## Results

Out of the total 290 participants who were included in the study, 146 (50.3%) were males and 144 (49.7%) were females. The number of patients who presented with a hypertensive crisis was 140 (48.3%), while 150 (51.3%) did not have a hypertensive crisis at presentation. 195 (79.6%) patients were obese, and 50 (20.4%) were non-obese, and data for obesity were missing on 45 patients since they could not be collected. The prevalence of depression was 143 (49.3%), while 160 (55.2%) of the total population had symptoms consistent with anxiety. The baseline clinical characteristics and sociodemographics of all our patients are summarized in Table 1.

**Table 1:** Sociodemographic and clinical characteristics of the study population (n=290) *'Hypertensive crisis’, described as systolic blood pressure greater than or equal to 180 mm Hg or diastolic blood pressure greater than or equal to 120 mm Hg. *The denominators for some variables are different because of missing data.

Characteristics	Overall	Patients with hypertensive crisis*	Patients without hypertensive crisis	P-value
	*N(%)	n(%)	n(%)	
	290(100)	140(48.3)	150(51.7)	
Age (years)				0.929
21–45	50(17.4)	26(18.7)	24(16.1)	
46–60	96(33.3)	45(32.4)	51(34.2)	
61–75	104(36.1)	49(35.3)	55(36.9)	
76–95	38(13.2)	19(13.7)	19(12.8)	
Sex				0.721
Male	146(50.3)	72(51.4)	74(49.3)	
Female	144(49.7)	68(48.6)	76(50.7)	
Marital status				0.676
Married	232(80)	113(48.7)	119(51.3)	
Never married	13(4.5)	5(3.6)	8(5.3)	
Widowed	41(14.1)	21(15)	20(13.3)	
Employment status				0.908
Yes	84(29)	41(29.3)	43(28.7)	
No	206(71)	99(70.7)	107(71.3)	
Education level				0.956
None	38(13.1)	20(14.3)	18(12)	
Primary	58(20)	26(18.6)	32(21.3)	
Secondary	95(32.8)	46(32.9)	49(32.7)	
College/university	92(31.7)	45(32.1)	47(31.3)	
BMI (kg/m^2^)				0.572
Obese	195(79.6)	84(80.8)	111(78.7)	
Non-obese	50(20.4)	20(19.2)	30(21.3)	
Duration of HTN				0.105
Less than 10 years	115(61.8)	57(60.6)	88(62.4)	
10-20 years	54(29)	28(29.8)	36(25.5)	
20-30 years	14(7.5)	12(9.5)	9(6.4)	
More than 30 years	3(1.6)	1(0.8)	8(5.7)	
Diabetes mellitus	153(52.9)	74(53.2)	79(52.7)	0.923
Chronic kidney disease	57(20)	28(20.3)	29(19.7)	0.906
IHD	78(27.2)	36(26.1)	42(28.2)	0.689
Cerebrovascular disease	31(10.7)	15(10.8)	16(10.7)	0.973
Alcohol use	4(1.4)	2(1.4)	2(1.3)	0.950
Smoking				0.993
Non-smoker	226(78.7)	108(78.8)	118(78.7)	
Past	32(11.1)	15(10.9)	17(11.3)	
Current	29(10.1)	14(10.2)	15(10)	
HADS depression	143(49.3)	69(49.3)	74(49.3)	0.994
HADS anxiety	160(55.2)	83(59.3)	77(51.3)	0.174

Table 2 demonstrates the comparison of depression and anxiety according to age categories in patients with and without hypertensive crisis. In patients with hypertensive crisis, HADS depression was most prevalent in the 61-75 age category (39.2%). Similar results were seen for HADS anxiety; it was most commonly seen in subjects with hypertensive crisis in the 61-75 year age category (34.9%).

**Table 2:** Comparison of depression and anxiety according to age categories in patients with and without hypertensive crisis *'Hypertensive crisis’, described as a systolic blood pressure more than or equal to 180 mm Hg or a diastolic blood pressure more than or equal to 120 mm Hg. **The denominators for some variables are different due to missing data.

	Patients with hypertensive crisis*	Patients without hypertensive crisis	P-value
	**n=140(48.3)	n =150(51.7)	
HADS depression	69(49.3)	74(49.3)	0.711
21–45 years	9(13.0)	13(17.8)	
46–60 years	22(32.4)	18(24.7)	
61–75 years	27(39.1)	32(43.8)	
76–95 years	10(14.7)	10(13.7)	
HADS anxiety			0.709
21–45 years	12(14.6)	14(18.4)	
46–60 years	28(34.1)	21(27.6)	
61–75 years	29(34.9)	31(40.8)	
76–95 years	13(15.9)	10(13.2)	

In a comparison of gender, it was found that males and females with hypertensive crisis had an almost equal prevalence of HADS anxiety (49.4% in males versus 50.6% in females). A slightly higher prevalence of depression was seen in females with hypertensive crisis when compared to males. These results are shown in Table 3.

**Table 3:** Comparison of depression and anxiety according to gender in patients with and without hypertensive crisis *'Hypertensive crisis’, described as a systolic blood pressure greater than or equal to 180 mm Hg or a diastolic blood pressure greater than or equal to 120 mm Hg. **The denominators for some variables are different due to missing data.

	Patients with hypertensive crisis*	Patients without hypertensive crisis	P-value
	**n=140(48.3)	n=150(51.7)	
HADS depression			0.544
Male	31(44.9)	37(50)	
Female	38(55.1)	37(50)	
HADS anxiety			0.507
Male	41(49.4)	34(44.3)	
Female	42(50.6)	43(55.8)	

In univariate logistic regression, as shown in Table 4, depression was not found to be associated with hypertensive crisis (p-value = 0.994), while the association between anxiety and hypertensive crisis was also not statistically significant (p-value = 0.174).

**Table 4:** Association of depression and anxiety with hypertensive crisis: univariate logistic regression

Characteristics	Odds ratio (95% confidence interval)	P-value
HADS depression versus no depression	0.998 (0.630–1.582)	0.994
HADS anxiety versus no anxiety	1.4 (0.867–2.198)	0.174

## Discussion

We found from this study that there was no association between depression or anxiety with hypertensive crisis. On further stratification by age groups and gender, the association remained the same. Multiple previous studies have investigated the relationship between depression and hypertension. A metaanalysis estimated the prevalence of depression amongst hypertensive subjects to be 21.3% [25]. But to the best of our knowledge, there is a paucity of data on the association between depression and hypertensive crisis, including hypertensive urgency and emergency. Broadly, there are studies that have linked depression with uncontrolled hypertension, which is expected in patients with hypertensive crisis as well. Wang et al. reported an association of depression with uncontrolled hypertension in the primary care setting (n = 1856) [11] and we have previously reported from a case-control study from Karachi a similar association between uncontrolled hypertension and depression (Odds ratio = 2.02) [8]. However, in the current study, we found no association of depression or anxiety specifically with hypertensive crisis overall. Our findings are in contrast to the former 2 studies on the association between depression and uncontrolled hypertension. The reason for this could be that previous studies were not specifically comparing hypertensive crisis patients with those without it and included patients with controlled hypertension as well, as opposed to the current study, which included only patients with uncontrolled hypertension. Second, previous studies were multi-centered studies, whereas this study was a single-center study. Despite these limitations, our findings still have important implications as 49% of patients with hypertensive crisis have depression and 59% have anxiety, which highlights the need to conduct large, multicentered studies. It also reinforces the importance of focusing on symptoms of depression and anxiety when managing patients with hypertensive crisis.

The underlying mechanisms between depression or anxiety and hypertensive crisis are that the stress response precipitated by depression leads to overactivity of the nervous system and the hypothalamic-pituitary-adrenal axis, promoting the sequelae of hypertension [26]. Other factors attributing to their correlation include poor adherence to antihypertensives in clinically depressed individuals, lack of physical activity, and use of antidepressant drugs [26]. Anxiety is also known to activate the sympathetic nervous system as a stress response mechanism, leading to the release of neurotransmitters that cause tachycardia, increased inotropism, and vasoconstriction, ultimately impacting blood pressure levels [27].

The highest proportion of depression and anxiety in our cohort occurred in the elderly (60-75 age group), with or without hypertensive crisis, although it did not reach statistical significance. These findings substantiate the previous reports that the elderly population is more liable to suffer from depression [28,29]. Moreover, the prevalence of depression in the elderly population found in our study aligns with that reported in a previous population-based cross-sectional survey conducted in Karachi, Pakistan, where the prevalence of depression was found to be 40.6% [28]. The observed difference could be because of the different scales used to assess depression in the two studies. Our results are also consistent with those reported from other countries. In a study of the elderly population in South Africa (upper middle income), depression was seen in 40% of the 3840 subjects included [30], while 30.1% were found to have depression in rural Malaysia (upper middle income) [31]. However, in another study conducted in Vietnam (LMIC) on people 60 years and above, a much higher rate of 66.9% was seen [32]. Amongst the factors identified, the companionship of a spouse, support of children, and a greater number of male children have been identified as protective factors against depression in the elderly [28]. Moreover, the elderly who had a greater number of chronic illnesses and a higher number of daily medications, financial constraints, urinary incontinence, and spiritual non-fulfillment were more likely to be depressed than their peers without these problems [29].

There are several limitations in our study that need to be illustrated. It was a single-centered study that hindered the generalizability of our results. Moreover, the small sample size and a cross-sectional design instead of a case-control or cohort design are additional limitations. These could be addressed in a future large multi-centered study. The assessment of hypertensive crisis was based on limited readings in an emergency setting and cannot be separated from the effect of white coat hypertension.

## Conclusions

We found no association between depression or anxiety with hypertensive crisis, and the association is not affected by age group or gender. Our findings do, however, underscore the significance of addressing symptoms of depression and anxiety while managing hypertensive crisis since the prevalence of depression and anxiety in the hypertensive crisis group was found to be significantly high. Future, large multi-centered studies are required to study the link in greater detail.

## References

[1] Fuchs FD, Whelton PK (2020). High blood pressure and cardiovascular disease. Hypertension.

[2] Zhou B, Perel P, Mensah GA, Ezzati M (2021). Global epidemiology, health burden and effective interventions for elevated blood pressure and hypertension. Nat Rev Cardiol.

[3] Mills KT, Stefanescu A, He J (2020). The global epidemiology of hypertension. Nat Rev Nephrol.

[4] Siddique S (2020). Asian management of hypertension: current status, home blood pressure, and specific concerns in Pakistan. J Clin Hypertens (Greenwich).

[5] Ipek E, Oktay AA, Krim SR (2017). Hypertensive crisis: an update on clinical approach and management. Curr Opin Cardiol.

[6] (2018). Global, regional, and national disability-adjusted life-years (DALYs) for 359 diseases and injuries and healthy life expectancy (HALE) for 195 countries and territories, 1990-2017: a systematic analysis for the Global Burden of Disease Study 2017. Lancet.

[7] (2022). Global, regional, and national burden of 12 mental disorders in 204 countries and territories, 1990-2019: a systematic analysis for the Global Burden of Disease Study 2019. Lancet Psychiatry.

[8] Almas A, Patel J, Ghori U, Ali A, Edhi AI, Khan MA (2014). Depression is linked to uncontrolled hypertension: a case-control study from Karachi, Pakistan. J Ment Health.

[9] Andrade L, Caraveo-Anduaga JJ, Berglund P (2003). The epidemiology of major depressive episodes: results from the International Consortium of Psychiatric Epidemiology (ICPE) Surveys. Int J Methods Psychiatr Res.

[10] Almas A, Ghouse A, Iftikhar AR, Khursheed M (2014). Hypertensive crisis, burden, management, and outcome at a tertiary care center in Karachi. Int J Chronic Dis.

[11] Wang L, Li N, Heizhati M, Li M, Yang Z, Wang Z, Abudereyimu R (2021). Association of depression with uncontrolled hypertension in primary care setting: a cross-sectional study in less-developed Northwest China. Int J Hypertens.

[12] Calvi A, Fischetti I, Verzicco I (2021). Antidepressant drugs effects on blood pressure. Front Cardiovasc Med.

[13] Cooper A, Aucote H (2009). Measuring the psychological consequences of breast cancer screening: a confirmatory factor analysis of the Psychological Consequences Questionnaire. Qual Life Res.

[14] Bjelland I, Dahl AA, Haug TT, Neckelmann D (2002). The validity of the Hospital Anxiety and Depression Scale. J Psychosom Res.

[15] Hansson M, Chotai J, Nordstöm A, Bodlund O (2009). Comparison of two self-rating scales to detect depression: HADS and PHQ-9. Br J Gen Pract.

[16] Smith AB, Selby PJ, Velikova G, Stark D, Wright EP, Gould A, Cull A (2002). Factor analysis of the Hospital Anxiety and Depression Scale from a large cancer population. Psychol Psychother.

[17] Mumford DB, Tareen IA, Bajwa MA, Bhatti MR, Karim R (1991). The translation and evaluation of an Urdu version of the Hospital Anxiety and Depression Scale. Acta Psychiatr Scand.

[18] Higashi A, Yashiro H, Kiyota K (1996). [Validation of the hospital anxiety and depression scale in a gastro-intestinal clinic]. Nihon Shokakibyo Gakkai Zasshi.

[19] Petersmann A, Müller-Wieland D, Müller UA (2019). Definition, classification and diagnosis of diabetes mellitus. Exp Clin Endocrinol Diabetes.

[20] Shahjehan RD, Bhutta BS (2022). Coronary Artery Disease. https://pubmed.ncbi.nlm.nih.gov/33231974/.

[21] Xin X, He J, Frontini MG, Ogden LG, Motsamai OI, Whelton PK (2001). Effects of alcohol reduction on blood pressure: a meta-analysis of randomized controlled trials. Hypertension.

[22] Primatesta P, Falaschetti E, Gupta S, Marmot MG, Poulter NR (2001). Association between smoking and blood pressure: evidence from the health survey for England. Hypertension.

[23] Good DC (1990). Cerebrovascular Disease. https://pubmed.ncbi.nlm.nih.gov/21250219/.

[24] Jafar TH, Chaturvedi N, Pappas G (2006). Prevalence of overweight and obesity and their association with hypertension and diabetes mellitus in an Indo-Asian population. CMAJ.

[25] Li Z, Li Y, Chen L, Chen P, Hu Y (2015). Prevalence of depression in patients with hypertension: a systematic review and meta-analysis. Medicine (Baltimore).

[26] Fernald F, Snijder M, van den Born BJ, Lok A, Peters R, Agyemang C (2021). Depression and hypertension awareness, treatment, and control in a multiethnic population in the Netherlands: HELIUS study. Intern Emerg Med.

[27] Lim LF, Solmi M, Cortese S (2021). Association between anxiety and hypertension in adults: a systematic review and meta-analysis. Neurosci Biobehav Rev.

[28] Bhamani MA, Karim MS, Khan MM (2013). Depression in the elderly in Karachi, Pakistan: a cross sectional study. BMC Psychiatry.

[29] Ganatra HA, Zafar SN, Qidwai W, Rozi S (2008). Prevalence and predictors of depression among an elderly population of Pakistan. Aging Ment Health.

[30] Peltzer K, Phaswana-Mafuya N (2013). Depression and associated factors in older adults in South Africa. Glob Health Action.

[31] Khan A, Ab Manan A, Rohana S (2010). Depression among the elderly Malays living in rural Malaysia. Internet J Public Health.

[32] Dao AT, Nguyen VT, Nguyen HV, Nguyen LT (2018). Factors associated with depression among the elderly living in urban Vietnam. Biomed Res Int.

